# Spinodal decomposition: a new approach to hierarchically porous inorganic materials for energy storage

**DOI:** 10.1093/nsr/nwz217

**Published:** 2019-12-26

**Authors:** Seongseop Kim, Jinwoo Lee

**Affiliations:** Department of Chemical and Biomolecular Engineering, Korea Advanced Institute of Science and Technology (KAIST), Korea; Department of Chemical and Biomolecular Engineering, Korea Advanced Institute of Science and Technology (KAIST), Korea

Hierarchically inorganic porous materials exhibit porosity on two or more distinct length scales, and therefore combine the benefits of the different pore sizes, which is highly desirable in a variety of applications in catalysis, separation and energy storage [1]. The macropores provide large diffusion pathways for rapid mass transfer and reservoirs for mass-buffering, and mesopores and micropores provide a high surface area [2]. Conventional methods for fabrication of hierarchically macro- and mesoporous inorganic materials have combined macroscale sacrificial templates (e.g. foams, polymer beads and silica spheres) for macropores and block copolymer (BCP) self-assembly for mesopores [3]. However, these strategies rely on complicated and time-consuming multistep procedures, including the preparation of macro-templates. Recently, spinodal decomposition (SD) has been explored to provide a new and simple approach to producing hierarchically macro- and mesoporous materials [4]. In this perspective, we highlight the opportunities and challenges, and provide an outlook of SD-derived hierarchically porous materials for energy storage.

**Figure 1.**
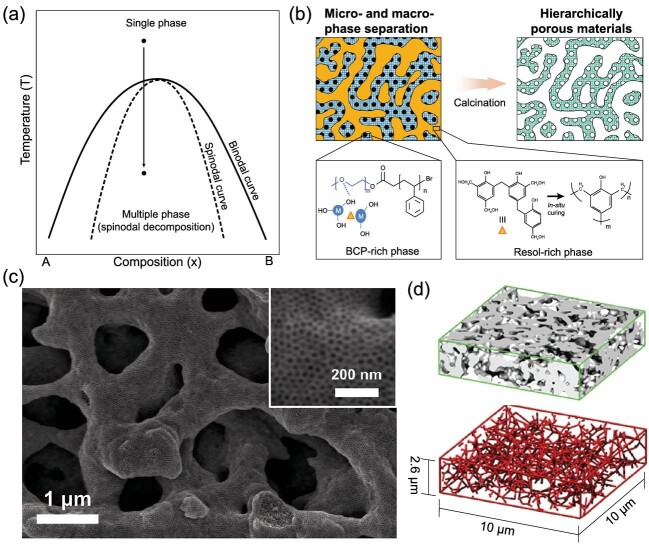
Schematic illustration of (a) phase diagram and (b) synthesis of hierarchically porous metal oxides by *in situ* curing of organic additive. (c) Scanning electron microscope (SEM) images of hierarchically macro- and mesoporous TiNb_2_O_7_. (d) Isosurface visualization of a 3D tomography obtained from reconstruction of nanoCT with a skeletal network of porous regions. (b) was modified with permission from Hwang *et al.* [11]; (c) and (d) were modified with permission from Jo *et al.* [14].

SD is a phase-separation process in which thermodynamic instability causes separation of homogeneous blends into two or more phases [5]. Unlike conventional phase-separation via nucleation and growth, SD evolves continuously by uphill diffusion within the blends without any nucleation process. The miscibility of a multicomponent blend can be changed by infinitesimal fluctuations (e.g. temperature change, chemical reaction and solvent addition or removal) under unstable supersaturated states. The small fluctuations reduce the free energy of the system and lead it into the spinodal region beyond the thermodynamic stability limit (${\partial ^2}G/\partial\! {X^2} < 0$); as a result, the system separates into bicontinuous two-phase macro domains (Fig. 1a). This macrophase separation of spinodally decomposed blends has attracted great attention for fabrication of hierarchically porous inorganic materials by combining microphase separation of BCPs [6–8].

In BCP-based blend systems with an organic additive (e.g. homopolymers and oligomeric precursors), the miscibility of blends is determined by the ratio r*_N_* (*N*_additive_/*N*_BCP_) of the degree of polymerization (DP) of the additive to DP of a block of the BCP. When r*_N_*${\rm{\ }} \ll $ 1, additive molecules are soluble with BCP, whereas at r*_N_*${\rm{\ }} \gg $ 1, macrophase separation occurs spontaneously [9]. Recently, Hwang *et al.* described a new approach to hierarchically macro- and mesoporous inorganic materials by exploiting these phase behaviors [10]. In multicomponent blends, including poly(ethylene oxide-*block*-styrene) (PEO_113_-*b*-PS_252_), high-molar-mass homopolystyrene (hPS_3360_) and inorganic precursor dissolved in organic solvent, large r*_N_* = 13 induces macrophase separation between (BCP/inorganic)-rich phase and hPS-rich phase as the solvent evaporates. PEO-*b*-PS simultaneously co-assembles with inorganic precursors to form mesostructures by microphase separation. Subsequent pyrolysis removes polymer species to leave inorganic materials with hierarchical porosity. Similarly, the DP increase of organic additives by chemical reaction can also induce spinodally decomposed macrophase separation [11]. When homogeneous PEO-*b*-PS/resol/inorganic precursor solution is used to achieve hierarchically porous metal oxides (e.g. SiO_2_ and TiO_2_), acid-polymerizable resol as an organic additive undergoes *in situ* curing by the increased acid concentration as solvent evaporates; this *in situ* curing leads to a large increase in the molar mass of additives (Fig. 1b).

**Figure 2.**
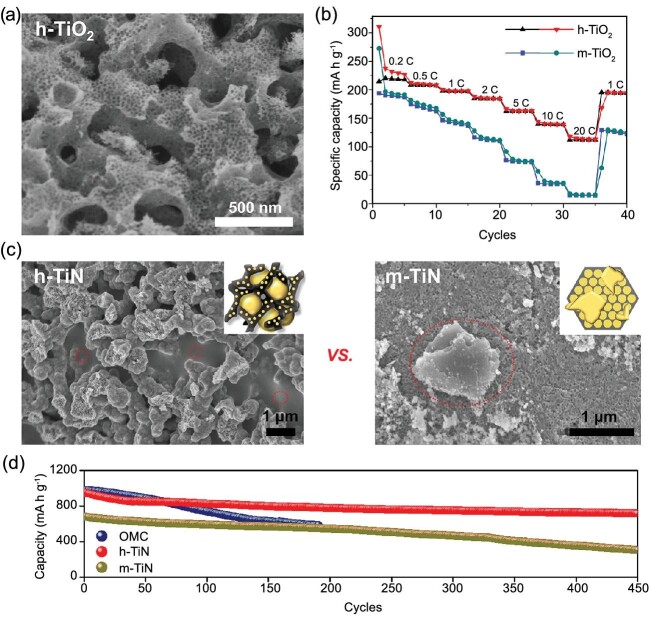
(a) SEM image and (b) the rate capability of h-TiO_2_ in LIB. (c) SEM images of h-TiN and m-TiN after sulfur impregnation. (d) Comparison of the cycle performance of ordered mesoporous carbon (OMC), h-TiN and m-TiN at 0.5 C rate in a Li–S battery. (a) and (b) were reprinted with permission from Hwang *et al.* [11]; (c) and (d) were modified with permission from Lim *et al.* [16].

The overpolymerized resol is immiscible with BCP and therefore is phase-separated from the BCP-rich phase, leading to bicontinuous macrodomains. BCP-directed self-assembly with inorganic precursors, followed by pyrolysis at 550°C in air, fabricates mesoporous metal oxide frameworks. These approaches using organic additives can independently control the macrostructures and mesopore size/structure by changing the amount of organic additives and BCP properties, respectively.

Macrophase separation by SD can also occur in solid–liquid phases. Sol–gel reaction with phase-separation by SD in titanium precursors/hPEO aqueous solution achieves macro- and mesoporous TiO_2_ monoliths [12,13]. However, mesopores are generated as interstices of nanocrystals, so this method cannot control the mesopore size and structures. Therefore, we explored BCP-based methods by using nitric acid and controlled evaporation rate, which provided a versatile synthesis platform for various metal oxides with macro- and mesopores [14,15]. A mixture of PEO-*b*-PS/precursors/nitric acid dissolved in tetrahydrofuran (THF) undergoes macrophase separation by the SD mechanism, and BCP self-assembly for mesopores. In this mixture, rapid solvent evaporation triggers macrophase separation by SD. Volatile THF quickly evaporates first and the remaining nitric acid phase becomes immiscible with the BCP/inorganic sol phase due to acid-derived inorganic sol–gel reaction. Fast solvent evaporation drives this unstable state to undergo SD with simultaneous BCP-inorganic co-assembly to yield macro- and mesoporous structures (Fig. 1c). On the other hand, slow solvent evaporation yields monolithic mesoporous materials without macropores because THF and nitric acid are removed simultaneously. This method can be used with various compositions (e.g. TiO_2_, WO_3_ and TiNb_2_O_7_) and hierarchically porous metal oxides can be easily transformed to metal nitrides [16]. Nanoscale X-ray computed tomography (nanoCT) exhibits homogeneous and co-continuous macroporous inorganic frameworks. The trivalent nodes of red skeletal networks indicates well-interconnected macropores (Fig. 1d).

Hierarchically porous materials have an ideal structure as electrode materials in rechargeable batteries: interconnected macropores facilitate ion/mass transfer; mesopores provide high surface area and shorten the diffusion length [17]. For instance, when used as an anode material in lithium ion batteries (LIB), hierarchically porous TiO_2_ (h-TiO_2_) had higher rate capability than mesoporous TiO_2_ (m-TiO_2_) (Fig. 2a and b) [11]. Furthermore, at 20 C rate, h-TiO_2_ had 52.6% capacity retention whereas m-TiO_2_ had only 8%. Although the physical properties of both TiO_2_ were almost identical except for macropores, the increased rate performance could be attributed to fast ion transfer via macropores. Similarly, it was also confirmed that macropores enhanced the rate performance when hierarchically porous TiNb_2_O_7_ was used as an anode material [14].

Macropores can also be beneficial as hosts for loading large amounts of active materials. In lithium–sulfur (Li-S) batteries, hierarchically macro- and mesoporous TiN (h-TiN) enables stable accommodation of large amounts of sulfur, and facile ion/electron transfer [16]. Macropores of h-TiN could confine 72 wt% of sulfur, whereas mesoporous TiN (m-TiN) could not accommodate the same amount, which instead aggregated on outside surfaces of m-TiN (Fig. 2c). These features of h-TiN improved the reversible capacity and the rate capability, and effectively prevented the shuttle effect of polysulfides (Fig. 2d).

Hierarchically porous structures with advantages of fast mass transfer and large pore volume can be a good inspiration to develop highly desirable inorganic materials for energy storage. The SD process combined with BCP self-assembly paves a new route for the fabrication of hierarchically macro- and mesoporous inorganic materials. The majority of SD-derived hierarchically porous inorganic materials are in bulk powder form with 3D macro-architectures, which could not be practical for all devices. Thus the tailoring of the macro-morphology at all levels from 3D to 0D, 1D and 2D is imperative for various applications yet still remains a great challenge. The future synthetic platforms for hierarchically porous materials should be able to control simultaneously both the pore size/structure and the morphology. A deeper understanding of the physics of multi-phase blends may provide insights for the synthesis of intriguing morphologies with pore hierarchy, which can beneficially impact energy storage devices and beyond.
